# Association between Visual Impairment and Low Vision and Sleep Duration and Quality among Older Adults in South Africa

**DOI:** 10.3390/ijerph14070811

**Published:** 2017-07-19

**Authors:** Karl Peltzer, Nancy Phaswana-Mafuya

**Affiliations:** 1 HIV/AIDS/STIs and TB (HAST), Human Sciences Research Council, Pretoria 0001, South Africa; nphaswanamafuya@hsrc.ac.za; 2 Department of Research & Innovation, University of Limpopo, Sovenga 0727, South Africa; 3 Office of the Deputy Vice Chancellor: Research & Engagement, Nelson Mandela Metropolitan University, Port Elizabeth 6001, South Africa; 4 Department of Social Work, University of Limpopo, Sovenga 0727, South Africa

**Keywords:** sleep duration, sleep quality, visual impairment, low vision, Study of Global Ageing and Adult Health, South Africa

## Abstract

This study aims to estimate the association between visual impairment and low vision and sleep duration and poor sleep quality in a national sample of older adults in South Africa. A national population-based cross-sectional Study of Global Ageing and Adults Health (SAGE) wave 1 was conducted in 2008 with a sample of 3840 individuals aged 50 years or older in South Africa. The interviewer-administered questionnaire assessed socio-demographic characteristics, health variables, sleep duration, quality, visual impairment, and vision. Results indicate that 10.0% of the sample reported short sleep duration (≤5 h), 46.6% long sleep (≥9 h), 9.3% poor sleep quality, 8.4% self-reported and visual impairment (near and/or far vision); and 43.2% measured low vision (near and/or far vision) (0.01–0.25 decimal) and 7.5% low vision (0.01–0.125 decimal). In fully adjusted logistic regression models, self-reported visual impairment was associated with short sleep duration and poor sleep quality, separately and together. Low vision was only associated with long sleep duration and poor sleep quality in unadjusted models. Self-reported visual impairment was related to both short sleep duration and poor sleep quality. Population data on sleep patterns may want to include visual impairment measures.

## 1. Introduction

“Globally, 285 million people are estimated to be visually impaired: 39 million are blind and 246 have low vision” [1], 90% of which live in developing countries and the majority (65%) with visual impairment are 50 years and older [1]. The most important causes of visual impairment include “uncorrected refractive errors (myopia, hyperopia or astigmatism) (43%), unoperated cataract (33%) and glaucoma (2%)” [1].

“Short sleep duration (<6 or <7 h per day) has been associated with a number of negative health outcomes, including diabetes mellitus, hypertension, cardiovascular diseases, coronary heart diseases, and obesity” [2]. Similarly, long sleep duration (>8–10 h) in the older adult population is associated with morbidity, including “hypertension, diabetes, atrial fibrillation, and poor general health” [3]. Moreover, poor sleep quality and insomnia are linked to developing diabetes and hypertension [4], cardiovascular disease [5], anxiety, and depression [6].

Only few studies investigated the correlation between visual impairment and sleep duration and poor sleep quality. In a national survey in the USA, visual impairment was related to both short (<6 h) and long (>8 h) sleep durations [7]. Among Korean adults, greater risk of measured visual impairment was found in the ≤5 h/night and ≥9 h/night groups, compared to the 7 h/night group [8]. Another study found that visually impaired older Americans had greater sleep disturbance [9]. Among Japanese, visually impaired individuals had more sleep-related problems compared to individuals who were not visually impaired [10]. Visual impairment was also found to be associated with anxiety, depression, and insomnia symptoms among Russian immigrants in New York [11].

To our knowledge, there have been no large population-based studies assessing the association between visual impairment and sleep duration and poor sleep quality in Africa. Our hypothesis is that visual impairment is associated with both short and long sleep durations and with poor sleep quality. The aim of this study was to investigate the association between visual impairment, low vision, sleep duration and poor sleep quality in a nationally representative sample of older South Africans.

## 2. Materials and Methods

### 2.1. Sample and Procedure

A cross-sectional population-based “Study of Global Ageing and Adult Health (SAGE)” involving a sample of 3840 persons (aged 50 years or older) was conducted in South Africa in 2008. A two-staged probability sampling strategy was used in order to provide precise national and sub-national estimates across strata, i.e., provincial level, geographic type (urban and rural), and population group (Black African**,** multi-ancestry (Coloured), Indian or Asian African, and White African). At the household level, trained nurses using a structured questionnaire interviewed all persons aged 50 years and older and health measurements were conducted; proxy respondents were identified for selected persons who were unable to complete the interview [12]. Post-stratification weights were generated to adjust for the age and sex population distribution in South Africa at the time of survey; more details can be found in [12]. The individual response rate was 77% [12]. The study protocol was approved by the “Human Sciences Research Council Research Ethics Committee (Protocol REC 5/13/04/06)” and the national Department of Health. Further, informed consent was obtained from study participants.

### 2.2. Measures

#### 2.2.1. Visual Impairment

Visual impairment was assessed with two questions: (1) Far vision: “In the last 30 days, how much difficulty did you have in seeing and recognizing a person you know across the road (i.e., from a distance of about 20 meters)?” [12]. (2) Near vision: “In the last 30 days, how much difficulty did you have in seeing and recognising an object at arm’s length (for example, reading)?” Response options were “none, mild, moderate, severe, and extreme/unable” [12]. Visual impairment was defined as having severe or extreme far and/or near vision difficulty.

#### 2.2.2. Low Vision

Using a tumbling “E” log MAR chart, visual acuity was assessed for both near and far vision in each eye [13]. Measured near and distance visual acuity was categorized into “low vision (0.01–0.25 decimal) and normal vision (0.32–1.6 decimal)” [14]. In this investigation, a study participant had low vision if he or she had either low near or far vision in both eyes.

#### 2.2.3. Sleep Duration

Two questions were posed about self-reported hours of sleep the previous night and the night before the previous night: “How many hours (and minutes) did you sleep?” [12]. The nocturnal sleep duration of the two days was averaged, and classified into ≤5, 6–8, and ≥9 h per day, following a similar categorization used in previous similar studies [7,8].

#### 2.2.4. Poor Sleep Quality

Poor sleep quality was assessed based on the question: “Overall in the last 30 days, how much of a problem did you have with sleeping, such as falling asleep, waking up frequently during the night or waking up too early in the morning?” (Response options ranged from 1 = none to 5 = extreme/cannot do) [12]. Poor sleep quality was defined as “severe or extreme/cannot do”.

#### 2.2.5. Covariates

##### *Age, Gender,* *Educational Level and Population Group*

Educational Level was assessed by asking participants, “How many years of school, including higher education, have you completed?” The response option was the number of years.

Economic or Wealth Status of a given household was estimated based on a list of household assets, and subsequently, wealth quintiles were created from these [15]. 

Chronic Diseases—such as angina, diabetes, and stroke—were measured by self-report, as having been diagnosed by a health care provider. 

Obesity, defined as having a body mass index (BMI) of 30.0 or higher kg divided by height metre squared [16], was measured by taking height and weight.

##### *Hypertension* 

Blood pressure (systolic and diastolic) was assessed “three times on the right arm/wrist of the seated respondent using an automated recording device (OMRON R6 Wrist Blood Pressure Monitor, HEM-6000-E, Omron Healthcare Europe, B.V., Hoofddorp, and The Netherlands)” [12]. Of the three blood pressure readings, the average of the last two readings was used [12]. Participants with “systolic blood pressure ≥ 140 mmHg and/or diastolic blood pressure ≥ 90 mmHg and/ or who reported the current use of antihypertensive medication” were classified as having high blood pressure [17].

##### *Depression* 

The World Mental Health Survey version of the “Composite International Diagnostic Interview” was used to assess symptom-based depression in the past 12 months [18]. The depression diagnosis was based on the “International Classification of Diseases” (ICD-10) [19], using an algorithm taking into account depressive symptoms in the past 12 months [20]. In addition, respondents who agreed to the question, “Have you been taking any medications or other treatment such as attending therapy or counselling sessions for depression during the last 12 months?” were added to the symptom-based diagnosis of depression.

The “World Health Organization (WHO) Disability Assessment Schedule (WHODAS-II)” was used to assess health, functioning, and disability in the past 30 days [21]. To estimate the severity of disability participants were asked about the level of difficulty with instrumental activities of daily living (IADLs) (ability to perform more complex tasks). Scores from the 12-item WHODAS-II are added up to get a total score, which is then converted to the following disability categories: “No problem (0–4%); Mild problem (5–24%); Moderate problem (25–49%); Severe problem (50–95%); Extreme problem (95–100%)” [21].

### 2.3. Data Analysis

Using STATA software version 13.0 (Stata Corporation, College Station, TX, USA) data were analysed taking into account the complex sampling design. Pearson correlation was administered to assess correlations between the different vision measures. Associations between confounding variables (socio-demographics and medical conditions) [7,8] visual impairment and low vision, short and long sleep duration, as well as poor sleep quality were assessed by calculating odds ratios (OR) using logistic regression (forced entry method). In the analysis, weighted percentages are reported. The *p*-values of less than 5% were used to indicate statistical significance. Both the reported *p*-values and the 95% confidence intervals are adjusted for the complex sample design of the study.

## 3. Results

### 3.1. Sample Characeristics

The total sample included 3840 individuals aged 50 years or older, with a mean age of 61.6 years (SD = 9.5), 44.1% men and 55.9% women and the most prominent population group was Back African (74.0%). Ten percent of the sample reported short sleep duration (≤5 h), 46.6% long sleep (≥9 h), 9.3% poor sleep quality, 8.4% self-reported visual impairment (near and/or far vision), 43.2% low vision (near and/or far vision) (0.01–0.25 decimal), and 7.5% low vision (near and/or far vision) (0.01–0.125 decimal). The proportion of poor sleep quality, visual impairment, and low vision was higher in older individuals, women and those with lower education. There was a correlation between visual impairment and low vision (0.01–0.025 decimal) (ρ = 0.07, *p* < 0.001) and low vision (0.01–0.125 decimal) (ρ = 0.09, *p* < 0.001) (see Table 1).

### 3.2. Associations Between Sleep Parameters and Vision

In fully adjusted logistic regression models, visual impairment was associated with short sleep duration and poor sleep quality, separately and together. Low vision (0.01–0.25 decimal) was only in an unadjusted model associated with long sleep and low vision (0.01–0.125) was only in an unadjusted model associated with poor sleep quality (see Table 2). Further, visual impairment was associated with short sleep (<7 h) and poor sleep quality (moderate to extreme) when adjusted for sociodemographic factors and chronic conditions, but became non-significant when additionally adjusted with severe limitations of instrumental activities of daily living (analysis not shown). 

## 4. Discussion

This study examined the association between visual impairment, low vision, sleep duration, and poor sleep quality in a nationally representative sample of older South Africans. Previous studies concentrated on studying these variables in high-income countries (Korea, Japan, and USA); the studies found that visual impairment was associated with the prevalence of short and long sleep duration and poor sleep quality [7,8,9,10,11]. This study only found an association between self-reported visual impairment and short sleep duration and poor sleep quality. Low vision was only found in unadjusted analyses associated with long sleep and poor sleep quality. The two different low vision measures (0.01–0.25 decimal and 0.01–0.125 decimal) showed both a weak correlation (<0.10) with self-reported visual impairment. This finding seems to indicate that only extreme visual impairment may lead to short sleep duration and poor sleep quality. Possible reasons for this could be that reduced light input due to low vision the suprachiasmatic nucleus (SCN) becomes incapable of adjusting to the day–night cycle, and sleep–wake cycles become desynchronized causing poor sleep quality [8,22]. In addition, “poor circadian entrainment and decreased exposure to daylight” could lead to early morning awakenings resulting in short sleep duration [7]. Furthermore, poor sleep quality and sleep apnea (a problem that causes sleep fragmentation) are common in individuals with visual impairment (often due to glaucoma) [7,23]. Moreover, persons with visual impairment may refrain from going outdoors since low vision may be a barrier to conducting outdoor activities leading to decreased exposure to bright light [8]. This may be supported by the finding of this study that after adjusting self-reported visual impairment with limitations of instrumental activities of daily living the previously significant association with short sleep (<7 h) and poor sleep quality (moderate to extreme) (analysis not shown) became non-significant. It has been shown that bright light treatment can be effective in treating insomnia in older adults [24]. In this study, more women than men were visually impaired, which seems congruent with previous surveys [25]. 

This study also had limitations. Since this was a cross-sectional study, no causative conclusions can be drawn between independent and outcome variables. Certain health variables such as sleep duration, certain chronic conditions, and visual impairment were assessed by self-report. However, Klein et al. [26] found a correlation between self-reported vision and measured visual acuity loss, with self-reporting severe visual difficulty corresponding to visual impairment (20/60 or 0.48 log MAR) [26].

## 5. Conclusions 

This study showed that self-reported visual impairment was associated with short sleep duration and poor sleep quality in a sample of older adults in South Africa. Health care providers should be aware of the possibility of sleep disorders in patients with visual impairment.

## Figures and Tables

**Table 1:** Sample characteristics, sleep pattern and vision

Variable	Sample	Sleep Duration			Poor Sleep Quality	Visual Impairment	Low Vision (0.01–0.25 Decimal)	Low Vision (0.01–0.125 Decimal)
	N (%)	≤5 h	6–8 h	≥9 h				
	M (SD)	M (SD)	M (SD)	M (SD)	M (SD)	M (SD)	M (SD)	M (SD)
**Sociodemographics**								
Age (years)	61.6 (9.5)	61.5 (10.0)	60.1 (8.7)	62.9 (9.7)	62.8 (9.9) *	64.1 (10.7) *	62.6 (9.7) *	65.1 (10.3) *
	N (%)	N (%)	N (%)	N (%)	N (%)	N (%)	N (%)	N (%)
Gender								
All	3840	209 (5.0)	1704 (48.4)	1614 (46.6)	328 (9.3)	295 (8.4)	1563 (43.2)	317 (7.5)
Female	2202 (55.9)	118 (5.2)	928 (45.6)	977 (49.2)	213 (11.4) *	196 (11.1) *	948 (45.2) *	216 (8.5) *
Male	1638 (44.1)	91 (4.8)	776 (52.0)	637 (43.2)	115 (6.7)	99 (4.9)	615 (40.7)	101 (6.2)
Education								
<7 years	1688 (51.9)	74 (3.9)	639 (39.2)	853 (56.8)	168 (10.8) *	162 (10.3) *	736 (46.1) *	168 (8.5) *
8–11	1051 (32.2)	82 (7.0)	512 (54.3)	396 (38.7)	82 (9.1)	69 (6.0)	415 (40.3)	87 (7.7)
12 or more	415 (15.9)	22 (5.6)	245 (60.8)	119 (33.6)	12 (3.0)	15 (7.5)	143 (37.8)	21 (3.4)
Wealth								
Low	1482 (40.6)	48 (2.8)	520 (39.9)	773 (57.3)	132 (10.3)	118 (9.2)	597 (43.1)	129 (7.3)
Medium	731 (18.2)	43 (5.9)	337 (47.7)	310 (46.7)	62 (8.9)	68 (10.3)	312 (44.3)	71 (6.3)
High	1608 (41.2)	116 (6.7)	838 (57.1)	525 (36.2)	131 (8.4)	108 (6.7)	648 (43.1)	115 (8.1)
Residence								
Urban	2561 (64.9)	164 (4.1)	1259 (40.2)	961 (55.8)	205 (8.5)	196 (8.2)	1060 (43.1)	231 (7.9)
Rural	1276 (35.1)	45 (5.5)	444 (52.8)	652 (41.8)	123 (10.8)	97 (8.5)	502 (43.4)	86 (6.8)
Population group								
Black African	2053 (74.0)	71 (3.8)	792 (43.0)	1028 (53.1)	178 (10.0) *	149 (8.9)	793 (41.7)	143 (6.9)
White African	269 (9.3)	27 (9.8)	160 (64.0)	65 (26.2)	12 (4.4)	9 (5.1)	97 (45.5)	13 (3.0)
Mixed ancestry	655 (12.8)	44 (5.6)	353 (60.5)	243 (33.8)	48 (6.2)	73 (8.8)	338 (53.9) *	95 (14.0) *
Indian/Asian African	307 (3.8)	36 (14.3)	147 (47.1)	92 (38.5)	32 (8.6)	26 (9.3)	132 (42.6)	32 (6.5)
**Medical conditions**								
Obesity	1539 (46.7)	87 (46.1)	732 (48.1)	636 (45.8)	121 (9.2)	130 (10.6)	655 (51.3)	120 (45.1)
Diabetes	360 (9.2)	34 (10.6)	182 (10.6)	134 (7.7)	54 (11.9)	44 (17.8)	178 (9.7)	42 (8.6)
Hypertension	2842 (77.3)	150 (76.6)	1275 (77.0)	1264 (78.5)	229 (76.9)	218 (75.4)	1234 (79.6)	246 (74.6)
Stroke	139 (4.0)	9 (2.7)	59 (3.7)	66 (4.4)	18 (6.4)	19 (5.8)	69 (4.7)	18 (4.2)
Angina	219 (5.2)	22 (6.0)	96 (4.5)	88 (5.7)	36 (8.7)	30 (6.7)	98 (5.8)	23 (5.9)
Depression	160 (4.0)	34 (12.4)	66 (3.2)	52 (4.3)	46 (14.4)	15 (3.9)	69 (3.2)	14 (3.0)
**Instrumental Activity of Daily Living (severe)**	562 (18.2)	50 (28.2)	200 (13.0)	312 (22.6)	172 (28.0)	149 (25.9)	297 (52.8)	66 (21.3)

*p < 0.001.

**Table 2:** Associations between visual impairment, low vision and sleep duration and poor sleep quality

Variable	Visual Impairment	Low Vision (0.01–0.25 Decimal)	Low Vision (0.01–0.125 Decimal)
	Odds Ratio (95% Confidence Interval)	Odds Ratio (95% Confidence Interval)	Odds Ratio (95% Confidence Interval)
**Short sleep (<6 h) #**			
Model 1 (unadjusted)	2.47 (1.04, 5.85) *	1.18 (0.73, 1.91)	1.24 (0.49, 3.16)
Model 2 (adjusted for age, sex, education, income, population group, residence)	2.91 (1.01, 8.43) *	1.28 (0.81, 2.02)	1.35 (0.53, 3.44)
Model 3 (adjusted for SES, obesity, diabetes, hypertension, stroke, angina, depression)	3.51 (1.40, 8.85) *	1.38 (0.85, 2.24)	1.32 (0.50, 3.54)
Model 4 (adjusted for SES, chronic conditions and IADL)	2.73 (1.23, 6.04) *	1.37 (0.81, 2.34)	1.29 (0.50, 3.33)
**Long sleep (9 or more hours) #**			
Model 1 (unadjusted)	1.56 (0.95, 2.57)	1.35 (1.07, 1.71) *	0.91 (0.50, 1.58)
Model 2 (adjusted for age, sex, education, income, population group, residence)	1.44 (0.91, 2.27)	1.22 (1.00, 1.50)	0.83 (0.49, 1.42)
Model 3 (adjusted for SES, obesity, diabetes, hypertension, stroke, angina, depression)	1.26 (0.76, 2.07)	1.20 (1.00, 1.44)	0.86 (0.59, 1.46)
Model 4 (adjusted for SES, chronic conditions and IADL)	1.06 (0.69, 1.62)	1.18 (0.97, 1.43)	0.85 (0.49, 1.48)
**Poor sleep quality**			
Model 1 (unadjusted)	5.88 (3.81, 9.06) *	1.15 (0.77, 1.72)	1.85 (1.11, 3.08) *
Model 2 (adjusted for age, sex, education, income, population group, residence)	5.83 (3.32, 10.25) *	1.08 (0.68, 1.73)	1.42 (0.71, 2.82)
Model 3 (adjusted for SES, obesity, diabetes, hypertension, stroke, angina, depression)	3.84 (2.24, 6.57) *	1.01 (0.68, 1.49)	1.50 (0.73, 3.07)
Model 4 (adjusted for SES, chronic conditions and IADL)	2.83 (1.69, 4.76) *	1.00 (0.68, 1.49)	1.44 (0.69, 3.01)
**Short sleep (<6 h) and poor sleep quality**			
Model 1 (unadjusted)	3.93 (2.37, 6.50) *	1.29 (0.85, 1.98)	1.39 (0.60, 3.19)
Model 2 (adjusted for age, sex, education, income, population group, residence)	3.74 (2.33, 6.00) *	1.64 (0.99, 2.72)	1.31 (0.54, 3.18)
Model 3 (adjusted for SES, obesity, diabetes, hypertension, stroke, angina, depression)	4.31 (2.50, 7.40) *	1.26 (0.83, 1.93)	1.37 (0.54, 3.50)
Model 4 (adjusted for SES, chronic conditions and IADL)	3.02 (1.65, 5.50) *	1.25 (0.78, 1.99)	1.33 (0.58, 3.02)

# 6–8 h sleep duration was the reference group; IADL = Instrumental Activities of Daily Living; * significant.
